# Biochemical Origin of Raman-Based Diagnostics of Huanglongbing in Grapefruit Trees

**DOI:** 10.3389/fpls.2021.680991

**Published:** 2021-08-19

**Authors:** Tianyi Dou, Lee Sanchez, Sonia Irigoyen, Nicolas Goff, Prakash Niraula, Kranthi Mandadi, Dmitry Kurouski

**Affiliations:** ^1^Department of Biochemistry and Biophysicsw, Texas A&M University, College Station, TX, United States; ^2^Texas A&M AgriLife Research and Extension Center at Weslaco, Weslaco, TX, United States; ^3^Department of Biochemistry and Biophysics, Department of Biomedical Engineering, Texas A&M University, College Station, TX, United States; ^4^Department of Plant Pathology and Microbiology, Texas A&M University, College Station, TX, United States; ^5^Department of Biomedical Engineering, Texas A&M University, College Station, TX, United States

**Keywords:** Raman spectroscopy, HPLC, carotenoids, hydroxycinnamates, huanglongbing, plant biochemistry

## Abstract

Biotic and abiotic stresses cause substantial changes in plant biochemistry. These changes are typically revealed by high-performance liquid chromatography (HPLC) and mass spectroscopy-coupled HPLC (HPLC-MS). This information can be used to determine underlying molecular mechanisms of biotic and abiotic stresses in plants. A growing body of evidence suggests that changes in plant biochemistry can be probed by Raman spectroscopy, an emerging analytical technique that is based on inelastic light scattering. Non-invasive and non-destructive detection and identification of these changes allow for the use of Raman spectroscopy for confirmatory diagnostics of plant biotic and abiotic stresses. In this study, we couple HPLC and HPLC-MS findings on biochemical changes caused by *Candidatus Liberibacter* spp. (*Ca. L. asiaticus*) in citrus trees to the spectroscopic signatures of plant leaves derived by Raman spectroscopy. Our results show that *Ca. L. asiaticus* cause an increase in hydroxycinnamates, the precursors of lignins, and flavones, as well as a decrease in the concentration of lutein that are detected by Raman spectroscopy. These findings suggest that *Ca. L. asiaticus* induce a strong plant defense response that aims to exterminate bacteria present in the plant phloem. This work also suggests that Raman spectroscopy can be used to resolve stress-induced changes in plant biochemistry on the molecular level.

## Highlights

- Raman spectroscopy is coupled to high-performance liquid chromatography (HPLC) and HPLC-based mass-spectroscopy to reveal the biochemical origin of metabolic changes in citrus trees that take place upon Huanglongbing (HLB) infection. HLB causes an increase in p-coumaric acid and several flavones in the plant leaves and a decrease in the concentration of carotenoid lutein that are detected by RS.

## Introduction

Raman spectroscopy (RS) is a label-free, non-invasive, non-destructive spectroscopic technique that provides information about the chemical structure of analyzed specimens (Cardona, 1975). The Raman effect is based on inelastic scattering of photons by molecules that are being excited to higher vibrational or rotational states (Kurouski et al., 2015). RS is commonly used in food chemistry (Almeida et al., 2010), electrochemistry (Zeng et al., 2016), forensics (Kelly Virkler and Lednev, 2009; López-López et al., 2013), and materials science (Cantarero, 2015). It can be used to monitor changes in protein secondary structure (Kurouski et al., 2012), elucidate composition and origin of body fluids as well as gun-shot residues (Bueno and Lednev, 2013; Hager et al., 2018). During the last decade, several companies released portable Raman spectrometers. This allowed for the use of RS directly in the field for evaluation of plant health (Yeturu et al., 2016; Sanchez et al., 2019b,c, 2020a). This technological development sparked the interest of agronomists, plant pathologists, and plant biologists in utilization of this technology for analysis of plant health status.

The interest in agricultural applications of RS was further enhanced by a demonstration of Raman-based diagnostics of biotic and abiotic stresses. Several groups independently demonstrated that spectroscopic analysis of plant leaves and seeds could be used for confirmatory diagnostics of diseases, nitrogen, phosphorus, and potassium deficiencies as well as salinity stress in plans (Yeturu et al., 2016; Egging et al., 2018; Farber and Kurouski, 2018; Mandrile et al., 2019; Sanchez et al., 2019a,c, 2020b,c; Huang et al., 2020). Such diagnostics are based on changes in the biochemistry of the plant (Farber et al., 2019a). Moreover, it has been shown that RS could be used to identify pathogens on a species level (Egging et al., 2018; Farber and Kurouski, 2018). The potential of RS was further amplified by its coupling with chemometrics. The use of partial least square discriminant analysis (PLS-DA) and other supervised chemometric methods enabled quantitative prediction of plant diseases. On average, around 90% accurate detection and identification of plant biotic and abiotic stresses could be achieved by RS (Egging et al., 2018; Farber and Kurouski, 2018; Mandrile et al., 2019; Sanchez et al., 2019c, 2020b,c; Farber et al., 2020).

Huanglongbing (HLB) is a psyllid-vectored bacterial disease (Mcclean and Oberholzer, 1965; Capoor et al., 1967) that causes a substantial negative impact on the citrus production in Asia and Africa. Around a decade ago, HLB appeared in the Americas (Bové, 2006). The pathogen associated with this disease is currently uncultured (Tsai and Liu, 2000) *Candidatus liberibacter* spp. (*Ca. L. asiaticus*), a Gram-negative bacterium inhabits the plant phloem in uneven and variable titers, making it difficult to detect and control (Morgan et al., 2012). At early stages, trees exhibit no symptoms retaining bacteria inside the phloem. This often results in false-negative results in polymerase chain reaction (PCR)-based diagnostics of HLB. Symptomatic trees exhibit chlorotic appearance and mottling of leaves; premature drop and asymmetry of fruits (Lee et al., 2015). Relatively slow propagation of symptoms implies significant challenges in HLB management. Typically, farmers prefer not to remove neither asymptomatic nor symptomatic trees which facilitates uncontrollable disease proliferation. Our group recently showed that RS could be used to detect and identify HLB in early and late states of the infection (Sanchez et al., 2019c). Moreover, we demonstrated that, by using RS, HLB can be distinguished from nutrient deficiency and secondary diseases such as blight (Sanchez et al., 2019b). Our recent findings show that RS is far more sensitive than qPCR, the golden standard in pathogen diagnostics (Sanchez et al., 2020d). Further elucidation of biochemical origin of RS-detected metabolite changes will further expand its utility in digital faming.

Typically, researchers assign observed spectroscopic changes to a certain *Ca. L. asiaticus* of chemical compounds, such as carotenoids or polyphenols (Egging et al., 2018; Farber and Kurouski, 2018; Sanchez et al., 2019c, 2020b,c; Farber et al., 2020). However, correlative understanding about the exact molecular species that is detected by Raman-based disease diagnostics is limited. Such information can be obtained by comparing RS with high-performance liquid chromatography (HPLC) and mass spectroscopy-coupled HPLC (HPLC-MS) (Hijaz et al., 2013; Killiny and Nehela, 2017; De Moraes Pontes et al., 2020). In this study, we performed systematic comparison of RS and reported HPLC (Killiny and Nehela, 2017) and HPLC-MS (Hijaz et al., 2013)-based biochemical changes associated with HLB, otherwise known as citrus greening disease.

## Materials and Methods

### Plants

Leaves of HLB-positive grapefruits (*Citrus × paradisi* Macfad., Rio Red) without any visible signs of mechanical or insect damage were collected from the field at Texas A&M University-Kingsville Citrus Center, Weslaco, TX (26°10′00.1″N 97°57′27.7″W). In total, four field HLB-positive and four field HLB-negative trees were sampled and analyzed. We also collected leaves of HLB-negative trees from the greenhouse to ensure they were true negatives, and to compare their fingerprints with field healthy samples (Supplementary Figure 1). Spectroscopic analysis showed that the biochemistry of HLB-positive trees was drastically different from both healthy greenhouse-grown and field-grown trees, whereas the two healthy sets exhibited highly similar Raman fingerprints. For further comparisons, only field healthy and field HLB-positive trees were considered to keep any plant and environmental variable uniform.

### Isolation of DNA and Quantitative PCR (qPCR)

Total plant DNA was isolated from the same batch of leaf samples used for carotenoid isolation. Briefly, ∼200 mg tissue was homogenized in 2 ml screw-cap microcentrifuge tubes for 60 s at 5,000 rpm using a Precellys 24 homogenizer (MO BIO Laboratories, Carlsbad, CA, United States). Two steel BB air gun beads (Walmart Supercenter, Bentonville, AR, United States) were added to every tube to improve homogenization (Almeyda-León et al., 2001). The final quality and quantity of DNA was measured and determined by a NanoDrop 1000 Spectrophotometer (Thermo Fisher Scientific, Wilmington, DE, United States) and agarose gel electrophoresis.

Extracted DNA was used to perform qPCR using the CFX384 Real-Time PCR Detection system (Bio-Rad Laboratories, Inc., Hercules, CA, United States). The qPCR reaction mix contained 5 μl of iTaq universal SYBR Green Supermix (Bio-Rad Laboratories, Inc.), 0.4 μl of 10 μM forward primer RNR-F (5′-GGATAGTCCTGTTATTGCTCCTAAA-3′), 0.4 μl of 10 μM reverse primer RNR-R (5′-ACAAAGCAGAAATA GCACGACAA-3′), and 2 μl of plant DNA with an average DNA concentration of 45 μM; the total reaction volume was scaled to 10 μl. The qPCR cycling had one cycle at 95°C for 3 min followed by 39 two-step cycles each at 95°C for 15 s and 55°C for 30 s, and a final melting curve of 65–95°C for 5 s. *Ca. L. asiaticus* gene encoding β-subunit of ribonucleotide reductase (RNR) was amplified to detect *Ca. L. asiaticus* (Zheng et al., 2016). A citrus *GLYCERALDEHYDE-3-PHOSPHATE DEHYDROGENASE C2* (*GAPC2*) (Mafra et al., 2012) gene was used as the endogenous reference gene for the normalization of qPCR data. The *Ca. L. asiaticus* Ct (threshold cycle) values were normalized to the citrus housekeeping gene *GAPC2*. In order to differentiate healthy from HLB-positive samples, a *Ca. L. asiaticus* Ct cutoff value ≤ 28 was used (Supplementary Table 1).

### Carotenoid Extraction

Collected leaf samples were (∼150 mg) homogenized using a mortar and pestle. A 1.5 mL solution of chloroform and dichloromethane (2:1, v/v) was added to the homogenate and agitated on a thermomixer at 500 rpm at 4°C for 30 min. For phase separation, 0.5 mL of 1 M sodium chloride solution was added to the homogenate, mixed by inversion, and centrifuged at 5,000 g for 10 min. The aqueous and organic phases were collected in separate tubes. The aqueous phase was subjected to another round of separation by adding 0.75 mL of chloroform and dichloromethane (2:1, v/v), followed by centrifugation at 5,000 g for 10 min. The second organic phase was collected and pooled with the first batch and dried by the centrifugal evaporation method. Dried pellet was re-dissolved in 200 μL of methanol/tert-methyl butyl ether (MTBE) (60/40, v/v) prior to injection into HPLC.

### HPLC Analysis

Leaf extracts were analyzed by reversed phase HPLC using a Waters 1525 pump equipped with a Waters 2707 auto sampler and a Waters 2489 photodiode array detector (PDA). Carotenoids were separated on a reverse-phase C_30_, 3 μm column (250 × 4.6 mm) (Thermofisher, part number 075723) using mobile phases consisting of (A) methanol/water (95:5, v/v) and (B) MTBE. The gradient elution used with this column was 97% A and 3% B at 0–6 min with a linear increase of B to 100% at 20 min and was returned to initial conditions at 23 min. The column temperature was maintained at 20°C. The eluting peaks were monitored at 450 nm using PDA. Quantification was performed using Breeze software comparing peak area with standard reference curves.

### Raman Spectroscopy

Four Raman spectra were collected from a leaf on the adaxial side using a hand-held Resolve Agilent spectrometer. Spectral acquisition time was 1 s, laser power 495 mW; spectral baseline corrected was performed by the spectrometer software. In total, around 50 surface spectra were collected from both HLB-infected and healthy plants. The Raman spectra of chemical standards were collected using a home-built confocal Raman microscope equipped with a Nikon TE-2000U inverted microscope. Laser light (λ = 785 nm) was generated by a solid-state continuous wavelength laser (Necsel, NJ, United States) and directed toward a 50/50 beam splitter (Chroma, Marlborough, MA). The sample was illuminated though 20X dry Nikon objective (NA = 0.45). The scattered light was collected by the same objective and directed back to the beam splitter. Next, elastically scattered photons were filtered out by an LP02-785RE-25 long-pass filter (Semrock, NY, United States). Inelastically scattered photons were dispersed on a 600 groove/mm grating (blazed at 750 nm) in an IsoPlane SCT 320 spectrograph (Princeton Instruments, NJ, United States) and captured using PIXIS:400BR CCD (Princeton Instruments, NJ, United States). Prior Proscan II motorized stage H117P2TE (Prior, MA, United States) was used to control the XYZ position of the sample. All data were processed using GRAMS/AI 7.0 (Thermo Galactic, NH, United States).

### Statistical Analysis

Raman spectra were imported into MATLAB R2020a (Mathworks) for statistical analysis. Raman spectra were normalized based on overall intensity. Kruskal-Wallis one-way analysis of variance was used to determine the changes at observed bands. Kruskal-Wallis one-way analysis tests whether the median in a set of samples is significantly different from other classes. The null hypothesis of this test was that there was no significant difference at the band of interests. The significant level (α) was 0.05. The ANOVA also reported a 95% confidence interval for the true value of median for each compared group. The overlapping confidence intervals were conducted using MATLAB multcompare function, which by default uses Tukey HSD to evaluate group-to-group differences.

## Results and Discussion

### Raman-Based Analysis of HLB and Healthy Plants

**FIGURE 1:**
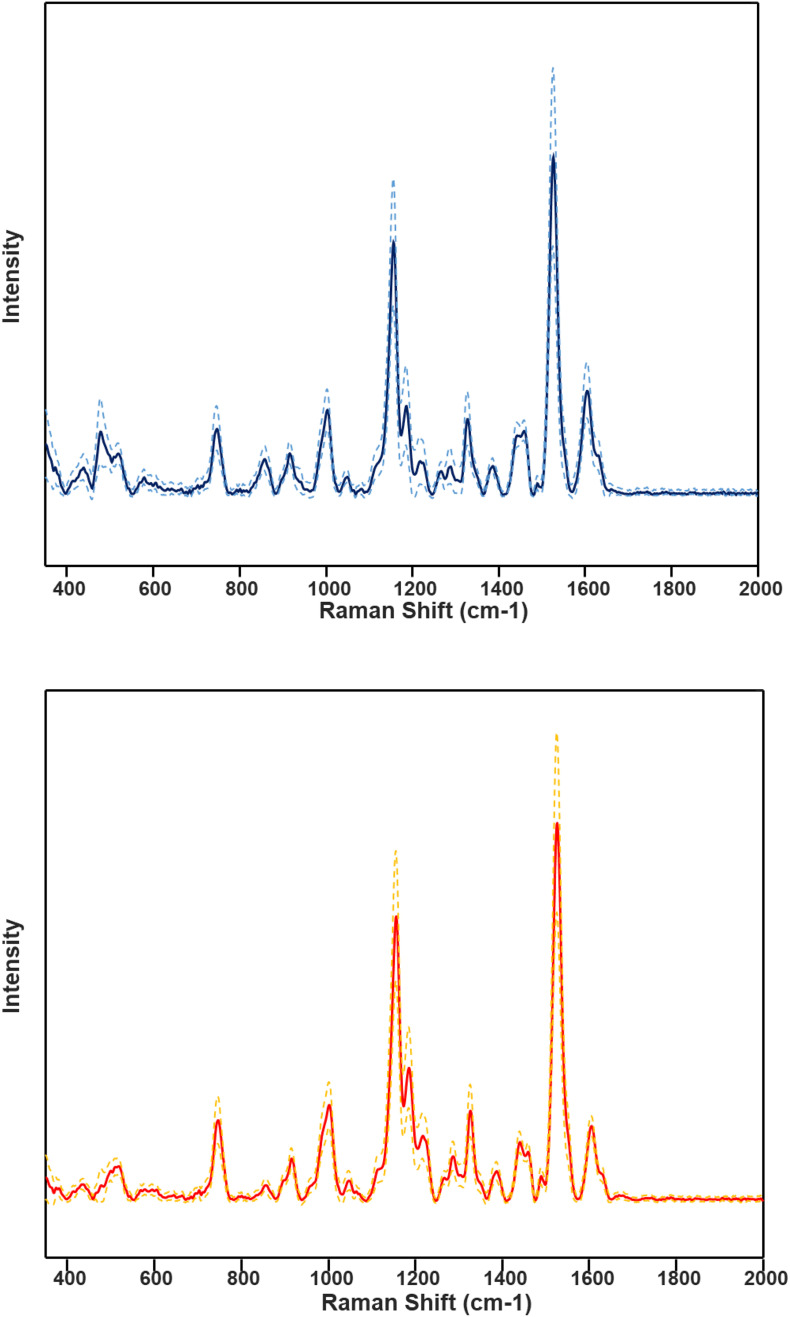
Average HLB spectrum (blue, top) with standard deviations (dashed lines) and healthy samples (red, bottom) with corresponding standard deviations.

**FIGURE 2:**
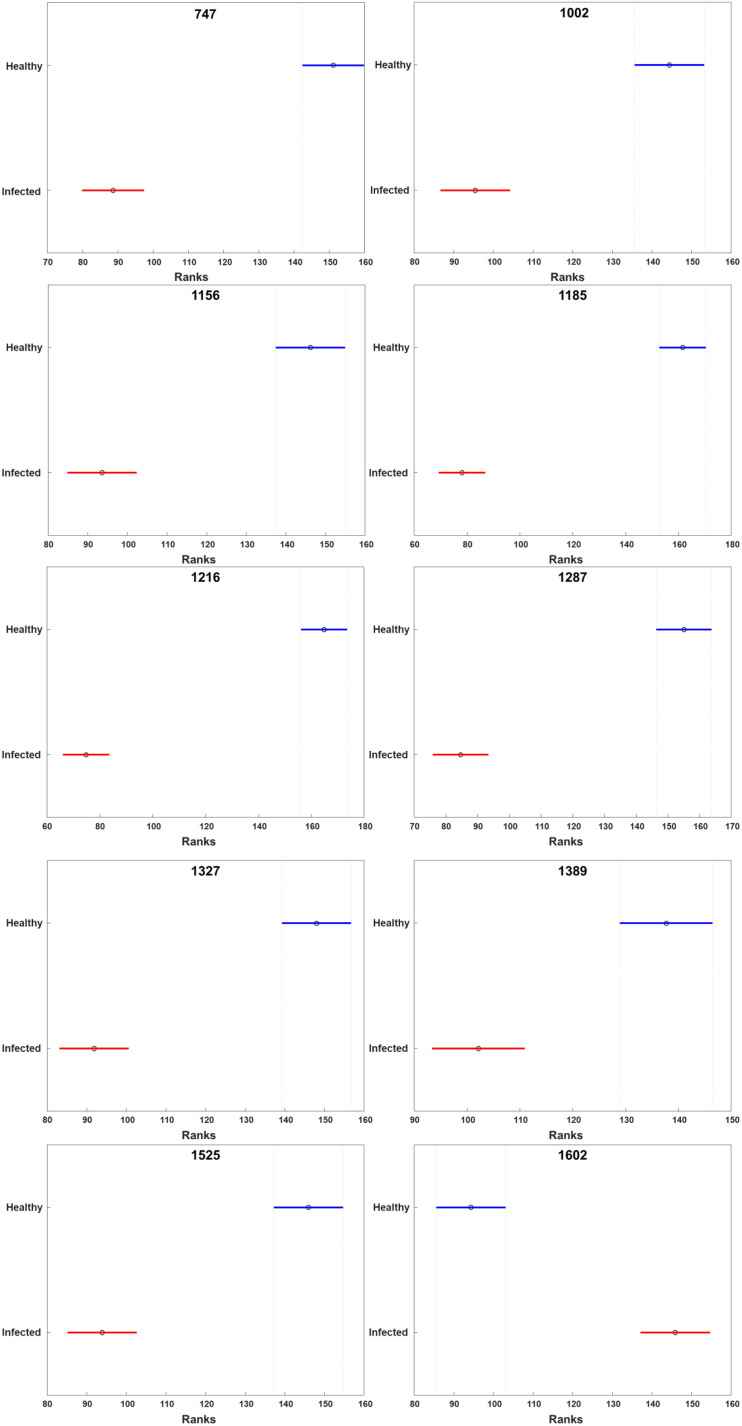
Kruskal-Wallis one-way analysis of variance results of all peaks observed in the Raman spectra of healthy and HLB-infected grapefruits. X axis stands for the rank of normalized intensity for each peak. The circle represents the median rank at the peaks. The line across the circle is the 95% confidence interval. The figure was generated by PLS_toolbox based on the Kruskal-Wallis results calculated by MATLAB.

HLB-associated changes in the spectra of citrus trees were in the vibrational bands centered at 1,000, 1,155–1,226, and 1,525 cm^–1^, as well as in 1,601–1,630 cm^–1^ (Figures 1, 2, Sanchez et al., 2019c). The last two bands originated from polyphenols, also known as phenylpropanoids, large *Ca. L. asiaticus* compounds that have aromatic moiety (Agarwal, 2006; Kang et al., 2016). It should be also noted that in the Raman spectrum collected from the leaves of healthy citrus trees, these two bands were centered at 1,606 and 1,630 cm^–1^, whereas in the spectrum of HLB-infected trees, the bands were found at 1,601 and 1,630 cm^–1^ (Figures 1, 2 and Supplementary Figure 2). HPLC-MS analysis of HLB-related changes on citrus trees reported by Hijaz and co-authors revealed an increase in the concentration of vitexin-2-O-rhaminozide (flavone), and hydroxycinnamates, also known as *p*-coumarates (Hijaz et al., 2013). All these *Ca. L. asiaticus* compounds are phenylpropanoids (Pompeu et al., 2018). Although an increase in the concentration of flavones was small, the concertation of hydroxycinnamates increased more than 10 times (Hijaz et al., 2013). Kruskal-Wallis one-way analysis of variance was conducted to normalized Raman spectra of HLB and healthy leaves. We observed a significant increase at bands at 1,602 cm^–1^ (Figure 2). This experimental evidence strongly suggests that an increase in the intensities of 1,601 and 1,630 cm^–1^ (Figure 1) is likely to be caused by an increase in the concentration of *p*-coumarates.

#### Determination of Spectroscopic Signatures of Molecular Analytes Detected by RS

**FIGURE 3:**
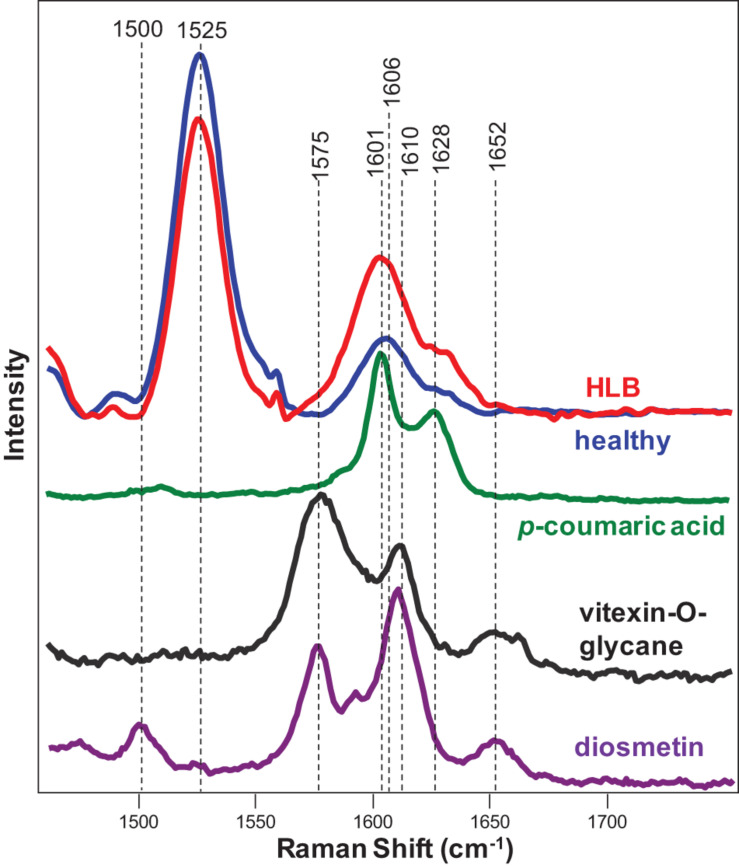
Raman spectra of healthy (blue) and HLB (red)-infected citrus leaves, as well as *p*-coumaric acid (green), vitexin-2-O-glucane, and diosmetin. Spectra from leaves were collected using a hand-held Raman spectrometer equipped with an 830 nm laser (*P* = 495 mW); spectra from chemicals were collected using a custom-built confocal Raman system using a 785 nm laser (13 mW).

Expanding upon this finding, we collected Raman spectra from pure standards of *p*-coumaric acid, as well as two flavones vitexin-2-O-rhaminozide and diosmetin (Figure 3). We found that *p*-coumaric acid had two peaks centered at 1,601 and 1,628 cm^–1^. These results confirm that an increase in the intensity of 1,601 and 1,630 cm^–1^ bands in the Raman spectra observed upon HLB infection was due to an increase in the concentration of *p*-coumaric acid/*p*-coumarates in the leaves.

The increase in the *p*-coumaric acid upon HLB infection is also supported by a study by Brodersen et al. (2014). Using microscopy analysis of leaves and stem sections of HLB-infected orange trees, the authors showed deformed cambium cells, collapsed phloem, and increased lignification of parenchyma cells. Indeed, *p*-coumaric acid is the precursor of H-lignin in plants. Thus, we can suggest that *Ca. L. asiaticus* infection triggers synthesis of *p*-coumarates that are further used for H-lignin biosynthesis. The lignification of cell walls likely is a plant defense response against the bacteria in attempts to limit their spread in the infected citrus tree.

In addition to increased intensities of 1,601 and 1,630 cm^–1^ bands, we observed the appearance of a small shoulder at 1,575 cm^–1^ in the Raman spectrum collected from the leaves of HLB-infected citrus trees. We also found that both vitexin-2-O-rhaminozide and diosmetin exhibited a 1,575 cm^–1^ band in their spectra (Figure 3). These results suggest that a shoulder at 1,575 cm^–1^ in the Raman spectrum collected from the leaves of HLB-infected citrus trees is likely to have originated from this flavone. Thus, we can suggest that an increase in the intensity of polyphenol vibrations in the Raman spectrum of HLB-infected trees is due to an increase in the concentration of two compounds: *p*-coumaric acid/*p*-coumarates and vitexin-2-O-rhaminozide and diosmetin.

**FIGURE 4:**
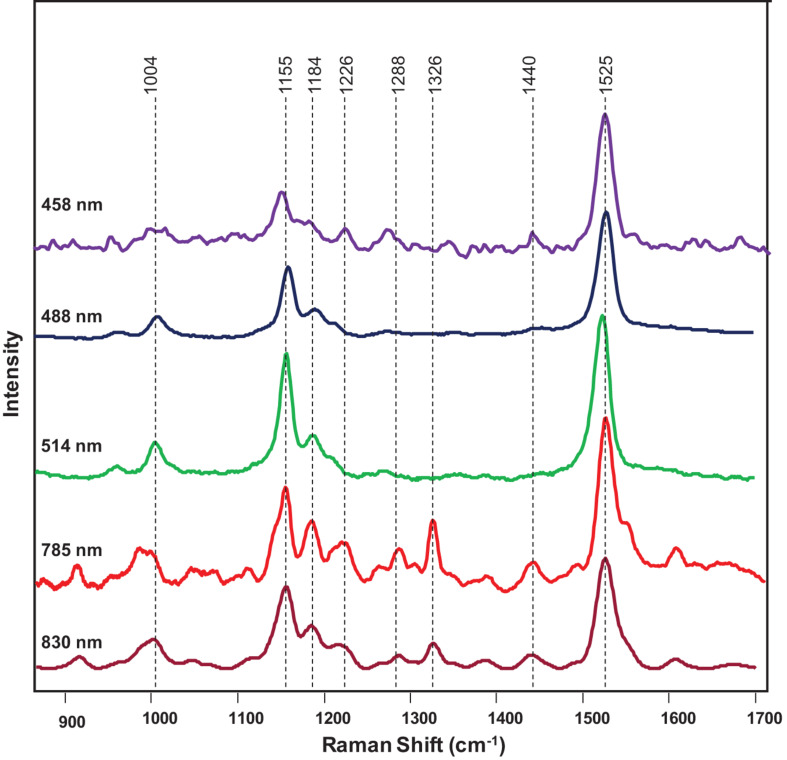
Raman spectra of a citrus leaf collected at 458 nm (purple), 488 nm (navy blue), 514 nm (green), 785 nm (red), and 830 nm (maroon).

Next, we investigated the underlying biochemical origin of the observed decrease in the intensities of 1,000, 1,155–1,218, and 1,525 cm^–1^ vibrations detected in the spectra collected from HLB-infected plants. The bands at 1,000 and 1,525 cm^–1^ can be assigned to different carotenoid species (Adar, 2017; Devitt et al., 2018), while the vibrations at 1,155, 1,184, and 1,218–1,226 cm^–1^ could be assigned to cellulose, xylan, or carotenoids (Edwards et al., 1997; Tschirner et al., 2009; Mary et al., 2012; Agarwal, 2014; Kurouski et al., 2015). It is known that carotenoids have absorption maxima in the blue-green part of the electromagnetic spectrum, while cellulose and xylan do not absorb light in that spectral region. Considering the differences in the absorption spectra of carotenoids and carbohydrates, we used a wavelength-scan approach in the blue-green range (λ = 458–514 nm) to achieve unambiguous assignment of 1,155, 1,184, and 1,218–1,226 cm^–1^ vibrations. Our results showed that, under the resonant Raman conditions (λ = 458-514 nm), all the bands at 1,005, 1,155, 1,184, 1,215–1,226, and 1,525 cm^–1^ could be readily detected in the spectra collected from plant leaves (Figure 4). This suggests that these vibrations originated from the same *Ca. L. asiaticus* biological molecules that are likely to be carotenoids.

#### Chromatographic and Spectroscopic Analysis of the Carotenoid Profile of Leaves

**FIGURE 5:**
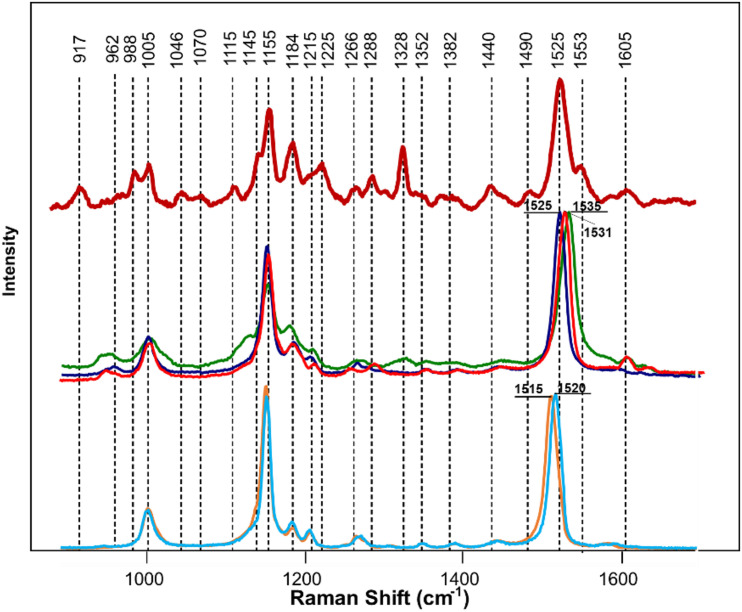
Raman spectra of a citrus leaf (maroon), lutein (blue), violaxanthin (red), neoxanthin (green), α-carotene (light blue), and β-carotene (orange). Leaf spectrum was collected using 830 nm laser excitation; spectra of chemical standards were collected using 785 nm laser excitation.

We next determined the different carotenoid species that could be assigned to these vibrations. HPLC results of carotenoid analysis in HLB-infected citrus plants performed by Killiny and Nehela revealed a significant decrease in the concentration of chlorophyll a and b, pheophytin a, chlorophyllide a, violaxanthin, neoxanthin, lutein, isolutein, and α and β-carotene comparing to healthy plants (Killiny and Nehela, 2017). Our HPLC results (discussed in S1) confirmed these findings (Supplementary Figure 2). Spectroscopic analysis of chlorophyll a revealed that this compound is highly auto-fluorescent and provided no meaningful Raman spectra. Similar results were obtained for its structural analog, chlorophyllin (data not shown). Structural similarity (presence of heme) between chlorophyll b, pheophytin a, and chlorophyllide a from one side and chlorophyll a and chlorophyllin from the other suggests that these compounds do not contribute to the Raman spectrum of the plant leaf. At the same time, Raman spectra of pure standards of α and β-carotenes, violaxanthin, neoxanthin, and lutein (Figure 5) exhibit similar band patterns to the carotenoid fingerprint of the leaf. We found that a vibrational band around 1,525 cm^–1^ can be used for identification of carotenoids and, therefore, can be considered as its “marker.” Specifically, α and β-carotenes exhibited this vibration at 1,520 and 1,515 cm^–1^, respectively. However, we found that in the Raman spectrum of the plant leaf, this band was centered at 1,525 cm^–1^. This suggests that neither α (1,520 cm^1^) and β-(1,515 cm^1^) carotenes are likely to contribute to the carotenoid signature of the plant leaf. The carotenoid marker was centered at 1,525 cm^–1^ in the Raman spectra of lutein, whereas in the Raman spectrum of violaxanthin and neoxanthin this band shifted to 1,531 and 1,535 cm^–1^ individually. Thus, it is likely that lutein is the major carotenoid contributor in the Raman spectrum of the plant leaf.

**FIGURE 6:**
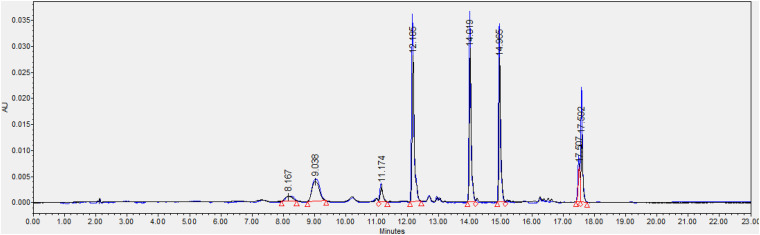
HPLC profiles of healthy (blue) and HLB-infected (black) grapefruit leaves.

**FIGURE 7:**
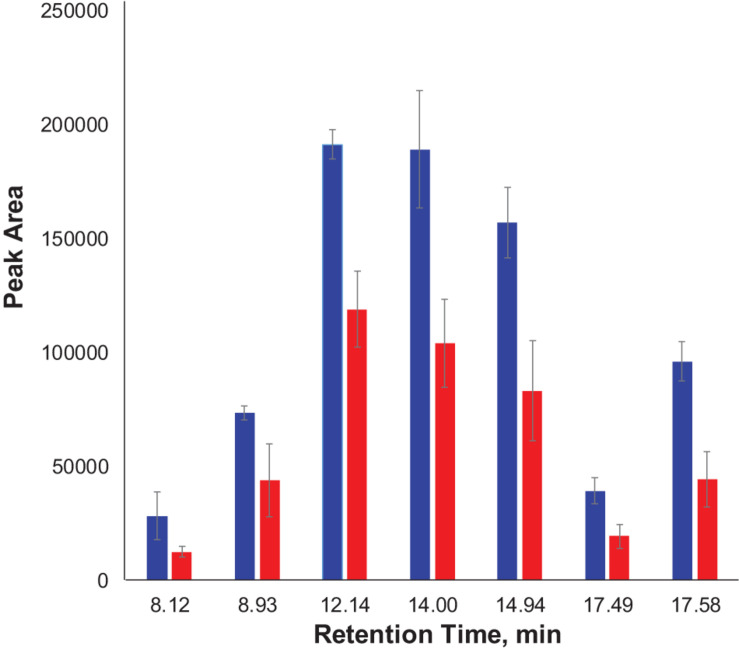
Average peak area and corresponding standard deviations of peaks observed in HPLC profiles of healthy and HLB-infected plants.

We also found that violaxanthin has a vibrational band at ∼1,605 cm^–1^ that was not evident in the spectra of other carotenoids. Since the spectroscopic fingerprint of HLB-infected leaves was associated with an increase rather than a decrease of this vibration at 1,605 cm^–1^ (as discussed above), violaxanthin is unlikely to be the predominant contributor of the carotenoid vibration in HLB plants and that leaves lutein as the primary carotenoid at 1,525 cm^–1^, that is lowered in HLB-infected leaves. HPLC analysis of grapefruit extracts performed in our laboratory confirmed a major decrease in the concentration of lutein in leaves of HLB-infected grapefruit trees compared to healthy plants (Figures 6, 7 and Table 1). This experimental evidence suggests that RS primarily detects a decrease in the concentration of lutein in plant leaves that take place upon HLB infection. Specifically, we observed a decrease in the concentration of lutein (RT = 12.185), chlorophyll (RT = 14.019), and α and β-carotene (RT = 17.507 and 17.562, respectively) in the leaves of HLB-infected plants comparing to healthy grapefruits (Table 2 and Supplementary Table 2).

**TABLE 1:** Two samples *t*-test results on bands of interests.

	**747**	**1,002**	**1,156**	**1,185**	**1,216**	**1,287**	**1,327**	**1,389**	**1,525**	**1,602**
H	1	1	1	1	1	1	1	0	1	1
*P*-value	2.6140e-05	3.5632e-08	2.8751e-09	6.5458e-05	0.0014	4.3651e-05	3.4297e-06	0.3716	1.3999e-08	2.1953e-10

**TABLE 2:** Chromatographic content of HLB-infected and healthy leaves.

**Retention time**	**Area**	**% Area**	**Height**
**HLB-infected leaves**			
8.437	27,125	8.14	876
11.025	16,429	4.93	1,420
12.12	101,949	30.58	14,369
14.000	80,566	24.16	18,123
14.954	57,923	17.37	14,565
17.504	14,055	4.22	3,921
17.589	35,366	10.61	8,633

**Healthy leaves**			
7.546	20,148	2.87	1,037
8.46	75,340	10.75	4,197
12.128	186,695	26.64	35,992
13.994	156080	22.27	35,965
14.953	136,928	19.54	33,854
17.505	31,475	4.49	8,904
17.591	94,237	13.45	22,483

A decrease in the carotenoid content revealed by both RS and HPLC in the case of HLB infection has strong physiological relevance to plant defense mechanisms (Havaux, 2013). Specifically, biotic and abiotic stresses activate enzymatic oxidation of neoxanthin that yields abscisic acid, a hormone that enhances plant resistance to such stresses (Nambara and Marion-Poll, 2005). β-carotene oxidation and cleavage by reactive oxygen species (ROS) lead to formation of β-lonone and β-cyclocitrals that can protect the plant against insects (Nambara and Marion-Poll, 2005; Havaux, 2013). Thus, reduction in this molecule could be a consequence of the higher ROS commonly triggered during plant defense reactions (Yu et al., 2007).

## Conclusion

Comparative analysis of RS and HPLC/HPLC-MS data of HLB-infected plants enabled characterization of molecular species whose levels were affected during citrus greening. We found that RS detects an increase in p-coumaric acid and several flavones, as well as a decrease in the concentration of lutein in HLB-infected leaves. Our findings suggest that RS can be reliably used to probe various biochemical changes occurring in plants during a defense response. The results of this work, as well as experimental findings reported by other research groups, suggest that RS can be used as a “fast diagnostics” approach that enables rapid screening of plant health (Yeturu et al., 2016; Altangerel et al., 2017; Farber and Kurouski, 2018; Farber et al., 2019b; Mandrile et al., 2019; Sanchez et al., 2019c, 2020b; Gupta et al., 2020; Huang et al., 2020). If more accurate identification of the pathogen strain or species is needed, a molecular methods of analysis, such as PCR, qPCR, or ELISA, can be used. Such a dual diagnostics approach is economically advantageous because RS requires no chemicals; it can be used directly in the field and the results are delivered within seconds. Thus, the use of RS can eliminate a large amount of work for more labor- and time-demanding molecular methods of analyses. We also envision that elucidation of the underlying biochemical determinants of Raman-based diagnostics of biotic and abiotic stresses should enhance the precision of RS. This may eventually eliminate the need for molecular methods of analysis in farming.

## Data Availability Statement

The raw data supporting the conclusions of this article will be made available by the authors, without undue reservation.

## Author Contributions

TD, NG, and LS collected spectra. PN performed qPCR analysis of samples. TD performed HPLC and chemometric analyses. SI and PN conducted citrus field surveys, RS sample collection, DNA isolation and qPCR diagnostics to determine HLB status/bacterial titers, and carotenoid extraction. TD, LS, PN, KM, and DK wrote the manuscript. All authors contributed to the submitted work.

## Conflict of Interest

The authors declare that the research was conducted in the absence of any commercial or financial relationships that could be construed as a potential conflict of interest.

## Publisher’s Note

All claims expressed in this article are solely those of the authors and do not necessarily represent those of their affiliated organizations, or those of the publisher, the editors and the reviewers. Any product that may be evaluated in this article, or claim that may be made by its manufacturer, is not guaranteed or endorsed by the publisher.

## Supplementary Material

Supplementary Figure 1 Average Raman spectra (solid lines) of HLB-infected plants (left) and healthy grapefruits (right) with corresponding standard deviations (dashed lines).

Supplementary Figure 2 Zoomed spectra of healthy and HLB-infected grapefruit tree leaves.

Supplementary Table 1 Quantitative real time PCR (qPCR) based diagnostics in healthy and HLB citrus leaves.

Supplementary Table 2 HPLC identification of carotenoids standard.
