# Supplementary material for: Biochemical Origin of Raman-Based Diagnostics of Huanglongbing in Grapefruit Trees
Source: Front Plant Sci. 2021 Aug 19;12:680991. doi: 10.3389/fpls.2021.680991 (PMC8417418; doi:10.3389/fpls.2021.680991)
Supplement: Supplementary Figure 1 — Average Raman spectra (solid lines) of HLB-infected plants (left) and healthy grapefruits (right) with corresponding standard deviations (dashed lines). [file Data_Sheet_1.docx]

Biochemical Origin of Raman-Based Diagnostics of Huanglongbing in Grapefruit Trees

Tianyi Dou^1^, Lee Sanchez^1^, Sonia Irigoyen^2^, Prakash Niraula^2^, Kranthi Mandadi^2,3*^ and Dmitry Kurouski^1*^

1. Department of Biochemistry and Biophysics, Texas A&M University, College Station, Texas 77843, United States
2. Texas A&M AgriLife Research and Extension Center at Weslaco, Texas 78496, United States
3. Department of Plant Pathology and Microbiology, Texas A&M University, College Station, Texas, 77843, United States

Supplementary Information

**Content:**

**Figure S1. Average spectra of healthy field-grown grapefruits (green dashed line), healthy greenhouse-grown plants (solid green line) and HLB positive trees (solid blue line).**

**Figure S2: Zoomed spectra of health and HLB-infected grapefruit tree leaves**

**Table S1: Quantitative real time PCR (qPCR) based diagnostics in healthy and HLB citrus leaves**

**Table S2: HPLC identification of carotenoids standard**

**Figure S1.** Averaged spectra of healthy field-grown grapefruits (green dashed line), healthy greenhouse-grown plants (solid green line) and HLB-positive field trees (solid blue line).

**Figure S2.** Zoomed 1400-1650 cm^-1^ region of healthy (green) and HLB-infected (red) grapefruit trees. Spectra are normalized on the intensity of 1601-1606 cm^-1^ for clarity.

**Table S1**: Typical quantitative real time PCR (qPCR) based diagnostics of field-grown healthy and HLB citrus leaf tissues. The Ct cut off value for HLB positive (Ct ≤ 28) and negative (Ct ≥30) are indicated.

| **Sample name** | **Raw Ct (RNR)** | **Ct normalized (RNR) to GAPC2** | **Diagnosis** | | |
| --- | --- | --- | --- | --- | --- |
|  |  |  | **Negative Cut off (≥30)** | **Borderline to cut off (28 to 30)** | **Positive Cut off (≤28)** |
| H1(healthy-1) | 33.84 | 34.01 | Negative |  |  |
| H2 (healthy-2) | 31.45 | 33.20 | Negative |  |  |
| H3 (healthy-3) | 32.18 | 33.20 | Negative |  |  |
| HLB1 | 23.98 | 23.79 |  |  | Positive |
| HLB2 | 25.71 | 25.71 |  |  | Positive |
| HLB3 | 23.01 | 23.00 |  |  | Positive |

**Table S2.** Identification of carotenoid standards.

| Retention Time | compound |
| --- | --- |
| 7.793 | neoxanthin |
| 9.107 | violaxanthin |
| 12.013 | lutein |
| 14.012 | chlorophyll |
| 17.494 | α-carotene |
| 17.591 | β-carotene |
